# Cancer-Related Constituents of Strawberry Jam as Compared with Fresh Fruit

**DOI:** 10.3390/cancers8010016

**Published:** 2016-01-14

**Authors:** Gema Flores, Maria Luisa Ruiz del Castillo

**Affiliations:** Instituto de Ciencia y Tecnología de Alimentos y Nutrición (ICTAN-CSIC), Consejo Superior de Investigaciones Científicas (CSIC), Juan de la Cierva 3, 28006 Madrid, Spain; gemaflo@hotmail.com

**Keywords:** strawberry jam, carcinogenic compounds, antitumor compounds, antioxidants, processing effects, heat induced compounds

## Abstract

The health awareness recently shown by consumers has led to a demand for health beneficial products. In particular, researchers are currently focusing their studies on the search for foods for cancer prevention activity. In the present work, we study comparatively the effect of two different processing methods on the contents of phenolic compounds (*i.e.*, ellagic acid, myricetin, quercetin and kaempferol) with antioxidant and antitumor properties in strawberry jams. In turn, the results obtained were compared with those of unprocessed fruit. Additionally carcinogenic heat-induced compounds formed by the two jam making methods were evaluated. Decreases of total ellagic acid from 138.4 µg/g to 86.5 µg/g were measured in jam as compared with the intact fruit. Even higher losses of up to 90% of total flavonols were found in strawberry after the jam-making process. A comparison between the two processing methods proved shorter heating periods (around 60 min) even at temperatures as high as 100 °C enabled losses of antioxidant phenolics to be minimized. Carcinogenic heat-induced volatile compounds, mainly Maillard reaction products, were formed as a result of thermal treatment during jam processing. However, shorter heating periods also helped reduce the formation of these harmful compounds. These results are deeply discussed. From a practical standpoint, the processing conditions here proposed can be used by industry to obtain strawberry jam with higher content of antioxidant flavonoids and, at the same time, reduced amounts of carcinogenic compounds.

## 1. Introduction

Because of the numerous health benefits associated with the intake of fruit, nutritionists recommend including fruits as a substantial part of our daily diet [1]. In particular, it is considered that fruits might help to reduce the risk for various types of cancer [2]. The potential biological effects of fruit constituents suggest plausible mechanism for protective effects, such as by reducing oxidative damage of DNA or increasing the activity of enzymes able to detoxify carcinogens [3]. However, fresh fruits are not available throughout the whole year and jams may be at times an alternative option to consume fruit ingredients all year round. In this context, for fruit jams to be commercially accepted, quality is essential. That involves that chemical composition of jams elaborated from fresh fruits must be carefully controlled to guarantee the preservation of naturally occurring bioactive compounds and assess, at the same time, the formation of carcenogenic compound resulting from processing.

Strawberry jam is one of the most popular jams and it represents a major economic interest in the food industry. Strawberry fruit is particularly known for its high content in ellagic acid and flavonol compounds, as well as for its pleasant aroma [4]. Ellagic acid is a phenolic compound with high antioxidant capacity [5]. It has been proven that ellagic acid induces apoptosis and possesses potential cytotoxic and anti-proliferative activities in human cells [6]. Similarly flavonols (e.g., myricetin, quercetin and kaempferol) have been reported to possess chemopreventive effects [7,8,9]. In particular, quercetin possesses additional health benefits such as anti-inflammatory [10] and anti-hypertensive properties [11].

Ellagic acid is present in strawberry in three different forms: the most common form, ellagitannin, (*i.e.*, esters of diphenic acid analogue with glucose), as ellagic acid glycoside and as free ellagic acid, the most unusual form [12,13]. Flavonols however occur in strawberry as *O*-glycosides, with a sugar bound at the C-3 position. All these forms are hydrolysable and, therefore, hydrolysis is the first step to be taken to determine the total contents of ellagic acid and flavonols in any foodstuff. Information on alterations produced in strawberry phenolics or volatiles as a result of jam processing is scarce in the literature. It is worthy to highlight the decline of total ellagic acid content [14] and the total flavonols [15] because of strawberry processing to obtain jams.

As far as strawberry aroma is concerned, it has been widely studied by many authors [16,17,18,19,20,21]. It is generally considered that a complex mixture of furanones, esters, aldehydes, alcohols, and sulphur compounds is responsible for the characteristic strawberry fruit aroma [22,23]. However, strawberry aroma is substantially modified by processing. In fact, the volatile compounds characteristic of fresh strawberries are mostly replaced in processed strawberry by certain heat-induced volatile compounds, such as isobutyraldehyde, furan, furfural and dimethyl sulphide, [24,25]. These compounds are a key source of flavour and colour and are responsible for the enjoyment of most heat processed foods, but some of them are also regarded as carcinogenic in such a way that their control is necessary [26].

This study was undertaken with the intention of assessing the contents of cancer-preventative constituents in strawberry jams elaborated from two different jam-making processes. In addition, constituents in both strawberry jams were compared with those in the fresh fruit. In particular, the study was focused on losses of natural antioxidants, which prevent cancer and related diseases, as well as on the formation of carcinogenic compounds due to strawberry processing. To that end, the composition of fresh strawberries, home-made strawberry jam and commercial strawberry jam were compared by keeping invariable all conditions except the time and temperature applied during processing.

## 2. Experimental Section

### 2.1. Samples and Materials

HPLC grade methanol (MeOH) and acetonitrile (ACN) were supplied by Labscan Ltd. (Dublin, Ireland). Trifluoroacetic acid (TFA), ellagic acid, myricetin, quercetin, kaempferol, morin and volatile standards were purchased from Sigma-Aldrich (Steinheim, Germany). Tert-butylhydroquinone (TBHQ) was provided by Fluka (Steinheim, Germany). Chloric acid (HCl) was purchased from Probus (Badalona, Barcelona). Milli-Q water was collected from a purification system (Millipore, Milford, MA, USA). The strawberry fruits used to elaborate home-made jam and the commercial strawberry jam included in this study were acquired from the local supermarket. For comparison, we used the same strawberry cultivar (Splendor variety, Egypt) and storage conditions (4 °C, 3 days) as those used by industry for commercial jam.

### 2.2. Strawberry Jam Homemade Preparation

Strawberry fruits (80 g) were chopped and slightly mashed (but not completely crushed) in a bowl. Then, water (200 mL) was added to the bowl and the contents were brought to a boil. The water/strawberry ratio was 2.5 (*v*/*w*). Subsequently the mixture was cooked to a full boil for 60 min. To make the jam as natural as possible, neither pectin nor sugar were added. We carefully selected fully ripe fruits in such a way that the amount of naturally occurring pectin was sufficient. Finally, the jam was allow to stand for 30 min before its analysis. The experimental conditions used for the home-made preparation were the same as those described for the industrial process. The phenolics and volatiles of the homemade jam were analyzed immediately.

### 2.3. Determination of Antioxidant Phenolic Content

#### 2.3.1. Extraction and Hydrolysis

The extraction and hydrolysis of ellagic acid, myricetin, quercetin and kaempferol from home-made and commercial strawberry jams was carried out as follows: a 20 g weight sample was homogenized with a blender. Acidified methanol (25 mL) containing 1% (*v*/*v*) HCl, 3.0 × 10^−3^ M TBHQ and the internal standard (morin, 0.5 µg) were added. Subsequently, HCl (1.2 M, 5 mL) was added to the mixture, which was then stirred at 90 °C under reflux for 2 h to hydrolyse glycosides to the corresponding aglycons. The resulting extract was allowed to get cold and then centrifuged at approximately 22,000 g for 10 min. The upper layer was taken, filtered through a 0.45 µm filter (Millipore) and analyzed by high performance liquid chromatography (HPLC) as explained below. For comparison, the extraction of the strawberry fruits was also carried out by following the same procedure.

#### 2.3.2. HPLC Analysis

Chromatographic analyses of the extracts were performed employing a model 560 liquid chromatograph (Konik-Tech, Barcelona, Spain) fitted with a manual injection valve (model 7725i, Konik-Tech) having a 20-µL sample loop and an ultraviolet (UV) detector operated at 360 nm. The simultaneous separation of ellagic acid, myricetin, quercetin and kaempferol was accomplished on a ODS reverse phase (C18) column (250 mm × 4.6 mm i.d., 5-µm particle size, ACE, Madrid, Spain). The elution was performed by using solvent A (H_2_O containing 0.1% TFA) and B (ACN/MeOH; 80/20) at a constant flow rate of 1.2 mL/min. A linear gradient was applied from the initial eluent composition, 70/30 (A/B, *v*/*v*), up to a final composition of 55/45 (A/B, *v*/*v*), which was reached at 30 min. Data acquisition was carried out by using Konikrom Plus (KNK-725-240). To identify ellagic acid, myricetin, quercetin, and kaempferol in the samples, standards were run under the same chromatographic conditions. Comparison between the retention times of the standards and those provided by the analytes were made. In addition, spiked extracts were also analyzed to confirm the identity of the target phenolics. Relative areas of ellagic acid, myricetin, quercetin and kaempferol with respect to that of morin were utilized to estimate their contents. The extraction and subsequent HPLC analysis of the extract were carried out in triplicate for each sample. The LC equipment was carefully washed by passing methanol through the whole system for 15 min after every single run. The analytical procedure here described including the extraction and HPLC analysis was equally applied to strawberry fresh fruits and both home-made and commercial strawberry jams.

### 2.4. Determination of Carcinogenic Heat-Induced Compounds

#### 2.4.1. Solid Phase Microextraction (SPME)

Approximately, 3 g of jam were weighed to carry out each analytical determination. The extraction of the heat-induced volatile compounds was performed by solid phase microextraction (SPME). A fused silica fiber coated with a 85-µm layer of carboxen/polydimethylsiloxane (CAR/PDMS) installed in a manual holder (Supelco, Madrid, Spain) was utilized. As recommended by the supplier, the fiber was conditioned in the injector of the gas chromatograph at 250 °C for 30 min before use. The strawberry jams were directly (with no previous manipulation) put in a 5.0 mL-vial. In both cases the vial was finally sealed. Prior to the actual extraction, the sample was left at 60 °C for 10 min for equilibration of the volatiles in the headspace. After the equilibration time, the SPME fiber was exposed to the sample headspace at 60 °C for 60 min. The extraction conditions applied were selected as a result of an optimization process in which distinct SPME fibres and extraction temperatures and times were tested (data not shown). When the extraction was completed, the compounds retained in the fiber were thermally desorbed by inserting the fiber into the injector port of the GC. Finally the volatile compounds were analyzed by gas chromatography-mass spectrometry (GC-MS), as specified below.

#### 2.4.2. GC-MS Analysis

A model 6890 gas chromatograph (Hewlett-Packard, Madrid, Spain) fitted with a split/splitless injector and mass spectrometry (MS) model HP5973 detector was employed to carry out the analyses. The SPME fiber was desorbed at 250 °C for 10 min into the GC injector. Splitless mode was used in all instances. GC separations were carried out on 25-m × 0.25-mm i.d. fused-silica column coated with a 0.25-µm layer of permethylated β-cyclodextrin (Chirasil-β-Dex, Chrompack, Whittier, CA, USA). The initial temperature of 40 °C was programmed at 5 °C/min to the final temperature (180 °C) which was maintained for 10 min. Helium was used as the carrier gas at a linear velocity rate of 1 mL/min and constant pressure mode was employed (10 psi). The source and the quadrupole temperatures were set at 230 °C and 100 °C, respectively. The SCAN mode was always used. Data acquisition from the MS was accomplished with the HP-ChemStation system. The identification of the volatile compounds analyzed was made by matching the mass spectra with those provided by the Wiley library. When doubt, the standards run in the same experimental conditions were used to verify the identities.

### 2.5. Statistical Analyses

Analysis of variance (ANOVA) of data on heat-induced compounds as well as on ellagic acid, myricetin, quercetin and kaempferol contents in strawberry was performed using JMP Statistics software package version 8 (SAS Institute Inc., Cary, NC, USA). The effect of jam elaboration process on the levels of carcinogenic heat-induced compounds and antioxidant phenolics was assessed by the Fisher test. Differences between data were compared by least significant differences (LSD). The values used were always the mean of the three replicates performed. Differences at *p* ≤ 0.05 were considered to be significant.

## 3. Results and Discussion

### 3.1. Determination of Antioxidant Phenolic Content

Table 1 shows the total contents of antioxidants, ellagic acid, myricetin, quercetin and kaempferol (µg/g fresh weight) in home-made and commercial strawberry jams. Data corresponding to unprocessed strawberries are also included for comparison. As can be seen in Table 1, the contents of all of them decreased significantly in both types of jams with respect to the intact fruit. In particular, myricetin diminished from 75.7 µg/g to 3.2 and 2.8 µg/g in home-made and commercial jams, respectively, and quercetin from 106.8 µg/g to 16.5 and 1.7 µg/g, respectively. These decreases represent approximately 90% lower contents of myricetin and quercetin in jams than in fresh berries. Total content of kampferol also declined significantly in both types of jams (from 26.8 µg/g to 10.5 and 7.8, respectively). This involves kampferol contents around 60% lower in jams than in berries. The decrease of total ellagic acid was not however so remarkable (from 138.4 µg/g to 107.0 and 86.5 µg/g, respectively). These values correspond to contents 23% and 38% lower in home-made and commercial strawberry jams, respectively, than those measured in fresh fruits. From these results it seems that flavonol compounds are more susceptible to losses during strawberry jam-processing than ellagic acid. By comparing jams to each other, the contents were always higher in the home-made elaboration than after the industrial processing, particularly for ellagic acid and quercetin (107.0 *vs.* 86.5 µg/g for ellagic acid and 16.5 *vs.* 1.8 µg/g for quercetin). Differences in contents between jams are associated with different conditions applied during the industrial and home-made jam-making processes. It is important to have in mind that vacuum is usually applied in industry to produce jam, which enables milder temperatures to be utilized. As a consequence, longer heating periods are usually required in industrial processes with respect to those needed in the home-made elaborations. Although little is known on the degradation behaviour of flavonols when foods undergo thermal processing, quercetin is believed to be degraded into protocatechuic acid or quinone, depending on the processing conditions [27]. No reports in this respect can be found in the literature on ellagic acid.

**Table 1:** Content (expressed as µg/g fresh weight ± standard deviation) of ellagic acid, myricetin, quercetin and kaempferol in strawberry fresh fruits and strawberry jams.

Samples	Ellagic Acid	Myricetin	Quercetin	Kaempferol
Fresh Fruit	138.4 ± 0.23 ^a^	75.7 ± 0.15 ^a^	106.8 ± 0.33 ^a^	26.8 ± 0.25 ^a^
Home-Made Jam	107.0 ± 0.21 ^b^	3.2 ± 0.11 ^b^	16.5 ± 0.13 ^b^	10.5 ± 0.11 ^b^
Commercial Jam	86.4 ± 0.17 ^b^	2.8 ± 0.08 ^b^	1.7 ± 0.10 ^b^	7.7 ± 0.08 ^b^

Data are presented as means (*n* = 3) ± SD where *n* refers to three independent samples; Different letters for the same compound in the same column between samples indicate differences at *p* < 0.05.

The results found here support data earlier published on the influence of processing conditions on onion flavonol glucoside [26]. These authors reported overall losses of up to 25% in quercetin glucoside in onions processed by two different forms of cooking. Equally, decreases in antioxidant flavonols of 14.8% for the kaempferol conjugates and 22.4% for the quercetin conjugated in canned beans with respect raw green beans have been described [28]. Particularly in berries, losses of flavonols in berries by cold-pressing, freezing and elaboration processes of juice and jams have been found [15]. These same authors have reported that strawberry industrial processing to produce jams decrease the total ellagic acid content in strawberries by 20% and flavonols by 15%–20% [14]. Amakura *et al.* [29] encountered losses of total ellagic acid content in raspberries by 16% in jam compared to the contents in fresh raspberries. The losses of ellagic acid and flavonols measured in the present study were far higher than 20%, which is probably the result of more extreme heating conditions used in the elaboration process.

In summary, significant losses of total ellagic acid and total myricetin, quercetin and kaempferol herein determined coincide with earlier bibliographic reports. To complete this study, the effect of jam processing on the content of flavonol derivatives might be interesting. It has been observed that processing and storage produce an increase in free ellagic acid of raspberries whilst ellagic acid glycosides are not affected [30]. This contrasts with the abovementioned drop in total ellagic acid after hydrolysis in processed strawberry products with respect to unprocessed strawberries. To our knowledge, no information on the contents of the flavonols myricetin, quercetin and kaempferol in this regard has been reported to date. Since ellagic acid monomers might be differently absorbed than high molecular weight ellagitannins, jam processing might not only affect the content of the antitumor compounds here studied but also their bioavailability. In this regard, although biological and bioavailability studies are required before coming to any conclusion, lower contents of total ellagic acid and total myricetin, quercetin and kaempferol might imply potential decrease in the antitumor properties of strawberry jam with respect to that of the intact fruit.

### 3.2. Determination of Carcinogenic Heat-Induced Compounds

**Figure 1:**
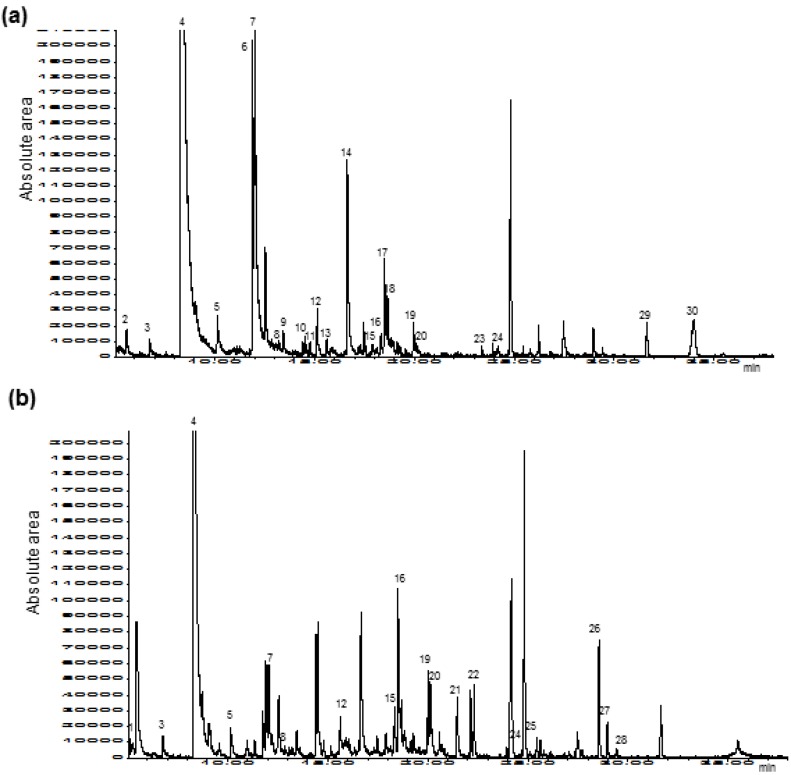
Chromatograms obtained from the SPME-GC-MS analysis of home-made (**a**) and commercial (**b**) strawberry jams under the best experimental conditions. The identification peaks were as follows: 1. 2-Methyl-1-penten-3-one, 2. Butyric acid, 3. Hexanal, 4. Furfural, 5. 2,5 dimethyl-4-methoxy-3-(2*H*)-furanone (mesifurane), 6. 5-Methylfurfural, 7. Benzaldehyde, 8. 2-Acetylfuran, 9. Acetophenone, 10. Nonanal, 11. Phenylacetaldehyde, 12. 2-Methylbutyric acid, 13. 2-Heptanone, 14. Methyl 3-hydroxyhexanoate, 15. Benzyl alcohol, 16. Hexanoic acid, 17. 2-Hexenyl hexanoate, 18. Ethyl 3-hydroxyhexanoate, 19. (−)-α-Terpineol, 20. (+)-α-Terpineol, 21. 2,4-Hexadienoic acid, 22. Isobutyric acid, 23. Furan, 24. 5-Hydroxymethyl-2-furfural, 25. Benzyl acetate, 26. 2,4-Bis(1,1-dimethylethyl)-phenol (BHT), 27. γ-butyrolactone, 28. Methyl 3-hydroxyoctanoate, 29. Farnesol, 30. (−)-Furaneol.

As far as heat-induced volatiles are concerned, Figure 1 illustrates the chromatograms obtained from the SPME-GC-MS analysis of home-made (a) and commercial (b) strawberry jams. Identifications were regarded as reliable when identified with 80% certainty by mass spectrometry and/or verified by standards. The repeatability was estimated from the Relative Standard Deviation (RSD, *n* = 5) for some selected major compounds. The RSD values obtained varied from 2.1% to 8.7% according to the specific compound considered.

For comparison chromatograms shown in Figure 1a and 1b were recorded at the same full range. All identified compounds in the jams have been previously described in the literature as processed fruit constituents [24,25,26,27,28,29,30,31]. Therefore, no unreported compound was detected in the present study. As observed in Figure 1, both compositional similarities and differences between the two jams were established. Among the similarities, we found hexanal, furfural, mesifurane, benzaldehyde, 2-acetylfuran, 2-methylbutyric acid, benzyl alcohol, hexanoic acid, both enantiomers of α-terpineol and 5-hydroxymethyl-2-furfural (HMF). These compounds were commonly identified in both jam samples whereas others however were determined as exclusive constituents of one or another type of jam. Specifically, butyric acid, 5-methylfurfural, acetophenone, nonanal, phenyl-acetaldehyde, 2-heptanone, methyl, 3-hydroxyhexanoate, 2-hexenyl hexanoate, ethyl 3-hydroxy-hexanoate, furan, farnesol and furaneol were exclusively detected in the home-made jam. In contrast, 2-methyl-1-penten-3-one, 2,4-hexadienic acid, isobutyric acid, benzyl acetate, BHT, γ-butyrolactone and methyl 3-hydroxy octanoate occurred only in the commercial jam.

To get an insight into semi-quantitative variations in the compounds commonly identified in both jams, absolute areas of volatile components were compared (Table 2). As depicted in the table, not only qualitative differences were observed between the two jams but also volatiles existing in both jams occurred in a very different proportion. In fact, the only compounds detected in comparable levels were hexanal and 2-methylbutyric acid. These two compounds are natural aroma-active compounds in many food plants. They are also additives permitted in food industry to reconstitute the fresh green odour of fruits lost during processing. Thus, their occurrence in similar proportions in home-made and commercial jams is not surprising. Either they naturally occur or they are deliberately added by industry.

**Table 2:** Absolute areas of volatile compounds identified in home-made and commercial strawberry jams by the SPME-GC-MS method developed and the experimental conditions selected (*i.e.*, CAR/PDMS fibre, 60 °C, 60 min).

Numbers	Compounds	Home-Made Strawberry Jam	Commercial Strawberry Jam
1	2-Methyl-1-penten-3-one	n.d. ^a^	6,060,266 ± 0.103
2	Butyric acid	798,148 ± 0.231	n.d.
3	Hexanal	667,617 ± 0.213 ^a^	600,082 ± 0.111 ^a^
4	Furfural	105,220,694 ± 0.105 ^a^	39,931,568 ± 0.206 ^b^
5	2,5-Dimethyl-4-methoxy-3-(2*H*)-furanone (mesifurane)	2,080,383 ± 0.182 ^a^	1,328,358 ± 0.232 ^b^
6	5-Methylfurfural	7,981,259 ± 0.098	n.d.
7	Benzaldehyde	18,199,786 ± 0.114 ^a^	2,818,282 ± 0.157 ^b^
8	2-Acetylfuran	1,132,193 ± 0.098 ^a^	25,701 ± 0.121 ^b^
9	Acetophenone	328,488 ± 0.214	n.d.
10	Nonanal	468,206 ± 0.036	n.d.
11	Phenylacetaldehyde	398,036 ± 0.213	n.d.
12	2-Methylbutyric acid	464,182 ± 0.086 ^a^	426,165 ± 0.145 ^a^
13	2-Heptanone	1,735,403 ± 0.201	n.d.
14	Methyl 3-hydroxyhexanoate	578,543 ± 0.193	n.d.
15	Benzyl alcohol	6,830,691 ± 0.231 ^a^	1 574,617 ± 0.254 ^b^
16	Hexanoic acid	521,249 ± 0.087 ^a^	5,347,177 ± 0.113 ^b^
17	2-Hexenylhexanoate	853,129 ± 0.151	n.d.
18	Ethyl 3-hydroxyhexanoate	3,201,276 ± 0.183	n.d.
19	(-)-α-Terpineol	717,332 ± 0.075 ^a^	1,854,972 ± 0.165 ^b^
20	(+)-α-Terpineol	983,539 ± 0.189 ^a^	2,165,148 ± 0196 ^b^
21	2,4-Hexadienoic acid	n.d.	813,206 ± 0.118
22	Isobutyric acid	n.d.	2,252,812 ± 0.126
23	Furan	717,332 ± 0.105	n.d.
24	5-Hydroxymethyl-2-furfural	683,539 ± 0.143 ^a^	6,528,233 ± 0.177 ^b^
25	Benzyl acetate	n.d.	414,871 ± 0.123
26	2,4-Bis (1,1-dimethylethyl)-phenol (BHT)	n.d.	2,501,944 ± 0.169
27	ɣ-Butyrolactone	n.d.	866,534 ± 0.088
28	Methyl 3-hydroxyoctanoate	n.d.	159,336 ± 0.092
29	Farnesol	451,790 ± 0.099	n.d.
*30*	(−)-Furaneol	6,019,384 ± 0.116	n.d.

^a^ Not detected; Data are presented as means (*n* = 3) ± SD where *n* refers to three independent samples; Different letters for the same compound in the same column between samples indicate differences at *p* < 0.05.

Regarding the volatiles existing mostly, or even exclusively, in home-made strawberry jam, naturally-occurring constituents predominated. Butyric acid, furfural, 5-methylfurfural, nonanal, 2-heptanone, farnesol, furaneol, furan, 2-acetylfuran, *etc*. are all natural constituents typical of fruits and plants although the formation by heating of some of them is also likely. In particular, furfural is at times formed as a product of carbohydrate caramelization and dehydration [32]. In the same way, the release of alcohols, such as farnesol, during the heat treatment of fruit juices has been frequently observed [33]. This phenomenon is attributed to the hydrolysis of the corresponding glycosidic precursors, such as fructose or glucose [33]. Furaneol is known worldwide as a one of the natural key aroma compounds in strawberry [34]. However, it is not so known that furaneol is unstable with pH and temperature [35]. Therefore it may result from the hydrolysis of its glucosidic precursors or as a Maillard reaction product [36,37]. Interestingly, furaneol was not even detected in the commercial sample, which reveals its natural origin and subsequent loss during commercial jam preparation. Furaneol was however preserved by the conditions applied during home made elaboration. On the other hand, although furaneol occurs in many foods and flavorants, it is also important to point out its toxicity with LD_50_ of 65 mg/kg (oral, rat) [38]. Not only natural constituents but also thermally formed compounds were detected in the home-made jam. For instance, benzaldehyde is generated from the thermal treatment as a result of the reaction of glucose with phenylalanine [37].

In contrast to home-made jam, volatile constituents prevailing, or even occurring as exclusive components, in the commercial strawberry jam were in general thermally induced compounds or additives usually added in jam industry. In particular, 2,4 hexadienoic acid (so-called sorbic acid) is employed in processed foods as a preservative. Also, BHT is industrially added as an antioxidant to guarantee the self life of the products. HMF is however, a Maillard reaction product that is formed by the reaction of glucose with glutamic acid [36,37,39]. Its characterization in blackberry juice subject to heating with sucrose has also been reported [33]. In fact, the concentration of HMF has been used for a long time as an indicator to monitor the intensity of changes taking place during thermal processing of foods. Its higher proportion in the commercial jam reflects the higher impact on volatiles of the longer processes used during the industrial processing. Besides, HMF has also been studied extensively as a food contaminant with potential carcinogenic properties [40,41]. Accordingly its occurrence in foods is undesirable. Other Maillard products are mesifurane and γ-butyrolactone. In fact, the higher influence on volatiles of the industrial jam-making process over the home-made elaboration was supported by the absence of γ-butyrolactone in the jam elaborated in the laboratory. γ-butyrolactone can be originated by thermal treatment [36,37,39] but also it can exist as a naturally occurring volatile compound [42]. When heat-induced, γ-butyrolactone has been reported to be formed either by glucose-glutamic acid Maillard reaction [38] or from the degradation of ascorbic acid [43]. Regardless its formation origin, safety of γ-butyrolactone for human health is currently doubtful. Studies on its impact on human health reflect possible carcinogenic effects as well as relation with mental changes, sedation, memory losses and heart problems [44].

In view of the results, it is believed that longer heating processes promote in a greater extent than higher temperatures the formation of harmful Maillard reaction products and, therefore the home-made jam processing is in this regard more advisable. Compounds resulting from heat processing affect the sensory, safety, nutritional and health-promoting attributed of foods [45]. Maillard products not only contribute to cancer risk but also influence the growth colonic bacteria and result in modifications of dietary protein. In addition to induction of Maillard reaction products, heating food is also associated with losses and reactions of indigenous heat labile components, such as polyphenols and vitamins.

## 4. Concluding Remarks

Overall, the home-made manufacturing of strawberry jam exhibited advantages over the industrial processing. Firstly, losses of antioxidant and antitumor compounds (*i.e.*, total flavonols and ellagic acid) were lesser by applying the home made process than the industrial processing. Secondly, natural-occurring strawberry aroma compounds were also more preserved in the home made jam. Finally, natural strawberry aroma compounds are mostly replaced by Maillard reaction products, most of them with carcinogenic properties, in the commercial jam. For these reasons, shorter heating periods whatever the temperature applied are proposed as an alternative to the industrial jam processing to obtain healthier and more pleasant product.

## References

[1] Van Duyn M.A., Pivonka E. (2000). Overview of the health benefits of fruits and vegetable consumption for the dietetics professional: Selected literature. J. Am. Diet Assoc..

[2] Bjelke E. (1975). Dietary vitamin A and human lung cancer. Int. J. Cancer.

[3] Steinmetz K.A., Potter J.D. (1991). Vegetables, fruit, and cancer. II. Mechanisms. Cancer Causes Control.

[4] Sharma S., Joshi V.K. (2009). An overview on strawberry [*Fragaria x ananassa* (Weston) Duchesne ex Rozier] wine production technology, composition, maturation and quality evaluation. Nat. Product. Rad..

[5] Losso N., Bansode R.R., Trappey A., Bawadi H.A., Truax R. (2004). In vitro anti-proliferative actitivities of ellagic acid. J. Nutr. Biochem..

[6] Narayanan B.A., Geoffroy O., Willinghanm M.C., Re G.G., Nixon D.W. (1999). *p53/p21(WAF1/CIP1)* expression and its possible role in G1 arrest and apoptosis in ellagic acid treated cancer cells. Cancer Letters.

[7] Sporn M.B., Liby K.T. (2005). Cancer chemoprevention: Scientific promise, clinical uncertainty. Nat. Clin. Pract. Oncol..

[8] Russo GL. (2007). Ins and outs of dietary phytochemicals in cancer chemoprevention. Biochem. Pharmacol..

[9] Orsolic N., Knezevic A.H., Sver L., Terzic S., Basic I. (2004). Immunomodulatory and antimetastatic action of propolis and related polyphenolic compounds. J. Ethnopharm..

[10] Elattar T.M.A., Virji AS. (2000). The inhibitory effects of curcumin, genistein, quercetin and cisplatin on the growth of oral cancer cells *in vitro*. Antic. Res..

[11] Pérez-Vizcaino F., Bishop-Bailley D., Lodi F., Duarte J., Cogolludo A., Moreno L., Bosca L., Mitchell J.A., Warner T.D. (2006). The flavonoid quercetin induces apoptosis and inhibits JNK activation in intimal vascular smooth muscle cells. Biochem. Biophys. Res. Commun..

[12] De Ancos B., González E.M., Cano M.P. (2000). Ellagic acid, vitamin C, and total phenolic contents and radical scavenging capacity affected by freezing and frozen storage in raspberry fruit. J. Agric. Food Chem..

[13] Häkkinen S.A., Heinonen M., Kärenlampi S., Mykkänen H., Ruuskanen J., Törronën A.R. (1999). Screening of selected flavonoids and phenolic acids in 19 berries. Food. Res. Internat..

[14] Häkkinen S.A., Kärenlampi S., Mykkänen H., Heinonen M., Törronen A.R. (2000). Ellagic acid content in berries: Influence of domestic processing and storage. Eur. Food Res. Technol..

[15] Häkkinen S.A., Kärenlampi S., Mykkänen H., Törronen A.R. (2000). Influence of domestic processing and storage on flavonol contents in berries. J. Agric. Food Chem..

[16] Schreier P. (1980). Quantitative composition of volatile constituents in cultivated strawberries, *Fragaria ananassa cv. Senga sengana, senga litessa and senga gourmella*. J. Sci. Food Agric..

[17] Hirvi T. (1983). Mass fragmentographic and sensory analysis in the evaluation of the aroma of some strawberry varieties. Lebensm Wiss & Technol.

[18] Latrasse A., Maarse H., Marcel D. (1991). Fruit III. Volatile Compounds in Foods and Beverages.

[19] Zabetakis I., Holden M.A. (1997). Strawberry Flavor: Analysis and biosynthesis. J. Sci. Food Agric..

[20] Schulbach K.F., Ruseff R.L., Sims C.A. (2004). Changes in volatile sulphur compounds in strawberry puree during heating. J. Food Sci..

[21] Jetti R.R., Yang E., Kurnianta A., Finn C., Qian M.C. (2007). Quantification of selected aroma-active compounds in strawberries by headspace solid-phase microextraction gas chromatography and correlation with sensory descriptive analysis. J. Food Sci..

[22] Pérez A.G., Olías R., Sanz C., Olías J.M. (1996). Furanones in strawberries: Evolution during ripening and postharvest shelf life. J. Agric. Food Chem..

[23] Pyysalo T., Honkanen E., Hirvi T. (1979). Volatiles of wild strawberries, *Fragaria vesca l.,* compared to those of cultivated berries, Fragaria ananassa cv Senga sengana. J. Agric. Food Chem..

[24] Sloan J.L., Bills D.D., Libbey L.M. (1969). Heat-induced compounds in strawberries. J. Agric. Food Chem..

[25] Barron D., Etiévant P.X. (1990). The volatile constituents of strawberry jam. Z. Lebensm. Unter. Forsch A.

[26] Ames J.M. (2009). Dietary Maillard reaction products: Implications for human health and disease. Czech. J. Food Sci..

[27] Makris D.P., ROSSITER J.T. (2000). Heat-induced, metal-catalyzed oxidative degradation of quercetin and rutin (quercetin 3-*O*-rhamnosylglucoside) in aqueous model systems. J. Agric. Food Chem..

[28] Price K.R., Bacon J.R., Rhodes M.J.C. (1997). Effect of storage and domestic processing on the content and composition of flavonol glucosides in onion (*Allium cepa*). J. Agric. Food Chem..

[29] Amakura Y., Umino Y., Tsuji S., Tonogay Y. (2001). Influence of jam processing on the radical scavenging activity and phenolic content in berries. J. Agric. Food Chem..

[30] Zafrilla P., Ferreres F., Tomás-Barberán F.A. (2001). Effect of processing and storage on the antioxidante ellagic acid derivatives and flavonoids in red raspberry (*Rubus idaeus*) jams. J. Agric. Food Chem..

[31] Sugawara E., Ito T., Odagirl S. (1982). Sweet aroma components in three kinds of jam. Nippon Nogeïkogaku Kaishi.

[32] Hodge J.E., Schultz H.W., Day E.A., Libbey L.M. (1967). Nonenzymatic Browning Reactions. Symposium of Foods: The Chemistry and Physiology of Flavors.

[33] Georgilopoulos D.N., Gallois A.N. (1987). Volatile flavour compounds in heated blackberry juices. Z. Lebensm. Unter. Forsch A.

[34] Mayerl F., Näf R., Thomas A.F. (1989). 2,5-Dimethyl-4-hydroxy-3(2*H*)-furanone glucoside: Isolation from strawberries and synthesis. Phytochemistry.

[35] Hodge J.E., Fisher B.E., Nelson E.C. (1963). Dicarbonyls, reductones, and heterocyclics produced by reactions of reducing sugars with secondary amine salts. Am. Soc. Brewing Chem..

[36] Hayase F., Kim S.B., Kato H. (1985). Maillard reaction products formed from d-glucose and glycine and the formation mechanisms of amides as major components. Agric. Biol. Chem..

[37] Mevissen L., Baltes W. (1988). Model reactions on roast aroma formation: VI. Volatile reaction products from the reaction of phenylalanine with glucose during cooking and roasting. Z. Lebensm. Unter Forsch. A.

[38] Rosatella A.A., Simeonov S.P., Frade R.F.M., Afonso C.A.M. (2011). 5-Hydroxymethylfurfural (HMF) as a building block platform: Biological properties, synthesis and synthetic applications. Green Chem..

[39] Berry S.K., Gramshaw J.W. (1986). Some new volatile compounds from the non-enzymic browning reaction of glucose-glutamic acid system. Z. Lebensm. Unter. Forsch. A.

[40] Hidalgo A., Pompei C. (2000). Hydroxymethylfurfural and furosine reaction kinectics in tomato products. J. Agric. Food Chem..

[41] Lee Y.C., Shlyankevich M., Jeong H.K., Douglas J.S., Surh Y.J. (1995). Bioactivation of 5-hydroxymethyl-2-furaldehyde to an electrophilic and mutagenic allylic sulfuric acid ester. Biochem. Biophys. Res. Comm.

[42] Habu T., Flath R.A., Mon T.R., Morton J.F. (1985). Volatile components of Rooibos tea (*Aspalathus linearis*). J. Agric. Food Chem..

[43] Tatum J.H., Shaw P.E., Berry R.E. (1969). Degradation products from ascorbic acid. J. Agric. Food Chem..

[44] National Toxicology Program (NTP) (1992). Toxicology and Carcinogenesis Studies of y-Butyrolactone (CAS No. 96-48-0) in F344fN1 Rats and B6C3F Mice (Gavage Studies), 1992.

[45] Motshakeri M., Ghazali H.M. (2015). Nutritional, phytochemical and commercial quality of Noni fruit: A multi-beneficial gift from nature. Trends Food Sci. Technol..

